# Ortner syndrome caused by aberrant right subclavian artery: A case report

**DOI:** 10.1097/MD.0000000000032272

**Published:** 2022-12-09

**Authors:** Yen-Wen Chen, Shih-Lun Chang, Nan-Chun Wu, Yun-Ju Shih

**Affiliations:** a Department of Otorhinolaryngology, Chi Mei Medical Center, Tainan, Taiwan; b Department of Pet Care and Grooming, Chung Hwa University of Medical Technology, Tainan, Taiwan; c Department of Cardiovascular Surgery, Chi Mei Medical Center, Tainan, Taiwan; d Department of Hospital and Health Care Administration, Chia Nan University of Pharmacy and Science, Rende, Taiwan; e Department of Medical Imaging, Chi Mei Medical Center, Tainan, Taiwan.

**Keywords:** aberrant right subclavian artery, hoarseness, Ortner syndrome, recurrent laryngeal nerve paralysis

## Abstract

Ortner syndrome (cardiovocal hoarseness) is characterized by recurrent laryngeal nerve paralysis secondary to a cardiovascular abnormality. Ortner syndrome caused by an aberrant right subclavian artery following a retroesophageal course without aneurysm formation is rare, with only 1 case reported in the literature. Cardiovascular abnormalities could be life-threatening and require early diagnosis and treatment. However, such abnormalities are not often considered by clinical practitioners when patients initially present with hoarseness.

**Patient concerns:**

A 34-year-old woman without any medical history presented to our outpatient department with hoarseness and mild dysphagia for 1 month.

**Diagnosis:**

Upon stroboscopic examination, left vocal cord incomplete paralysis was noted. Contrast-enhanced computed tomography revealed an aberrant right subclavian artery arising from the left aortic arch, causing focal compression of the esophagus and, potentially, compression of the left recurrent laryngeal nerve compression. The patient was diagnosed as left recurrent laryngeal nerve paralysis caused by an aberrant right subclavian artery following a retroesophageal course without aneurysm formation.

**Interventions:**

The patient was referred to a cardiovascular surgeon for resection and bypass surgery. Both the dysphagia and the hoarseness improved after the surgery.

**Outcomes:**

Significant improvement of the left vocal cord paralysis and no vocal cord adduction were seen upon stroboscopic examination after 3 months. During the 5-year follow-up period, the patient remained well, and no signs of recurrence were noted.

**Conclusion:**

This case can increase otolaryngologists’ awareness of this etiology of hoarseness and consider it in their differential diagnosis.

## 1. Introduction

The recurrent laryngeal nerve innervates intrinsic laryngeal muscles and thereby regulates the movement of the vocal cord. Injury of the recurrent laryngeal nerve often results from iatrogenic causes or from compression or invasion by benign or malignant tumors. Unilateral vocal cord paralysis may cause hoarseness and choking, and bilateral vocal cord paralysis may induce dyspnea. The cause of recurrent laryngeal nerve paralysis is rarely a cardiovascular abnormality.

Ortner syndrome (OS) is characterized by hoarseness caused by left recurrent laryngeal nerve paralysis secondary to a cardiovascular abnormality and is also referred to as cardiovocal syndrome. Hoarseness is the most common symptom, followed by dysphagia or dyspnea. Recurrent laryngeal nerve paralysis of the left side is 1.75 times more common than that of the right side because the left recurrent laryngeal nerve is longer than the right. Several cardiovascular abnormalities can cause OS^[1]^; however, only a few cases that are caused by an aberrant right subclavian artery have been reported.^[1,2]^ To our knowledge, only 1 reported case of OS was caused by an aberrant right subclavian artery without aneurysm formation^[2]^ and esophageal compression.^[1]^ Here, we present the case of a 34-year-old woman who experienced hoarseness and mild dysphagia and was diagnosed with left recurrent laryngeal nerve paralysis caused by an aberrant right subclavian artery after a retroesophageal course without aneurysm formation. Cardiovascular abnormalities can be life-threatening and should be considered by otolaryngologists in differential diagnoses for hoarseness.^[3]^ This case report highlights the rarity of our patient’s condition and the diagnostic process.

## 2. Case report

**Figure 1.**
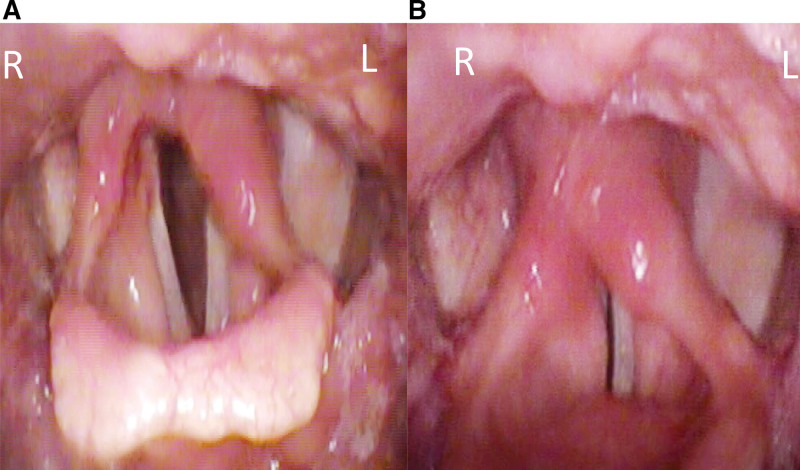
Incomplete paralysis of the left vocal cord.

**Figure 2.**
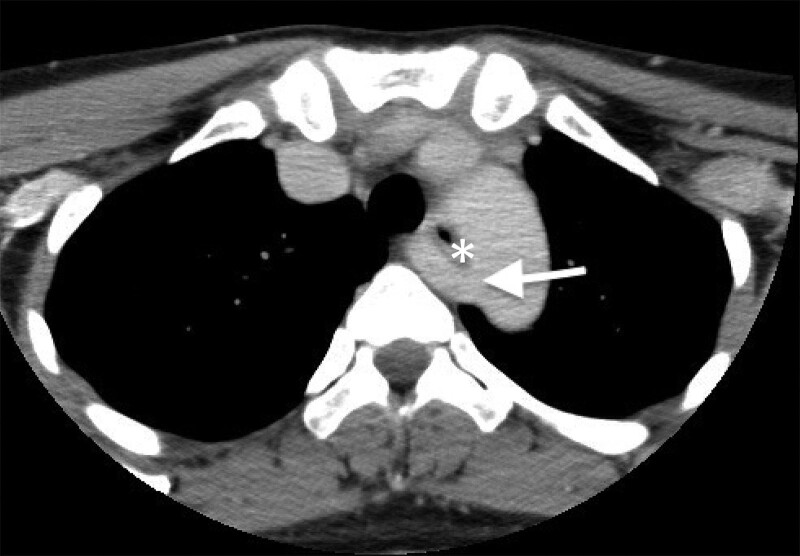
Contrast-enhanced computed tomography reveals an aberrant right subclavian artery (arrow) arising from the left aortic arch, which is located posterior to the esophagus (asterisk), suggesting a retroesophageal course.

**Figure 3.**
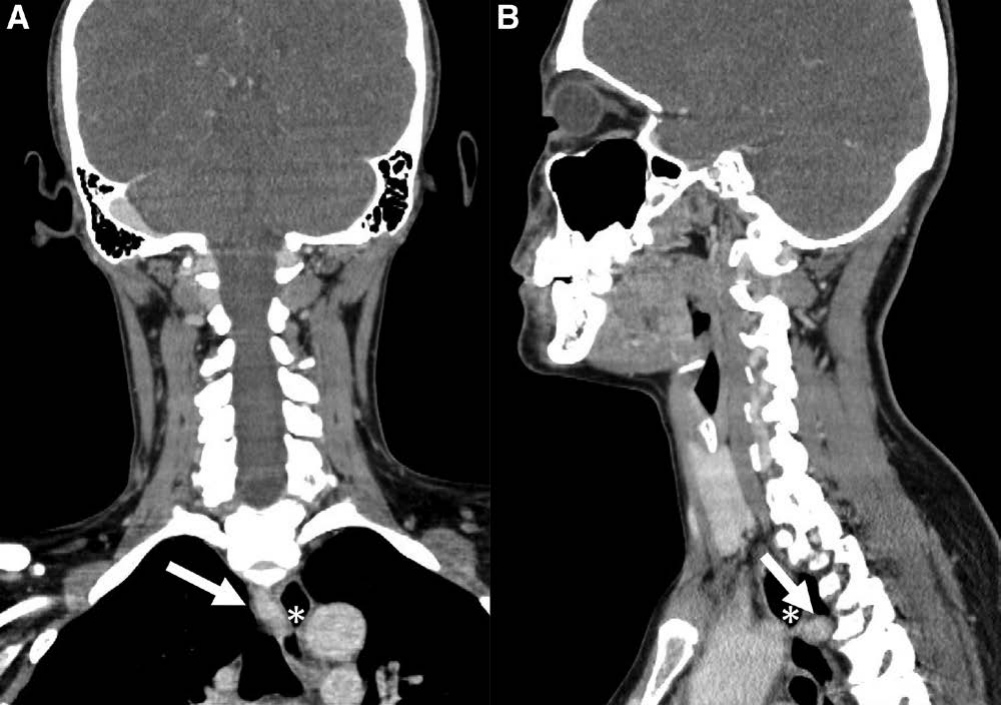
(A) Coronal and (B) sagittal contrast-enhanced computed tomography images show esophageal compression (asterisks) caused by the aberrant right subclavian artery (arrows).

**Figure 4.**
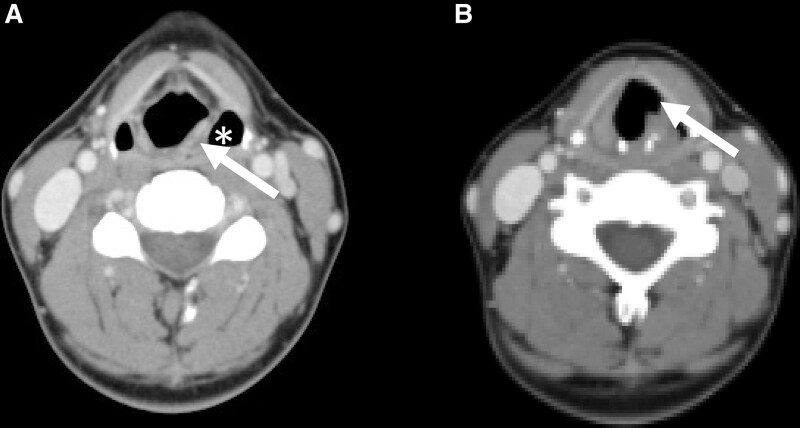
(A) Axial section of neck computed tomography image revealing dilatation of the left pyriform sinus (asterisk) and medialization of the left aryepiglottic fold (arrow). (B) Axial section of neck computed tomography image at the level of the vocal cord revealing dilatation of the laryngeal ventricle, also known as the sail sign (arrow). These features are consistent with left vocal cord paralysis.

**Figure 5.**
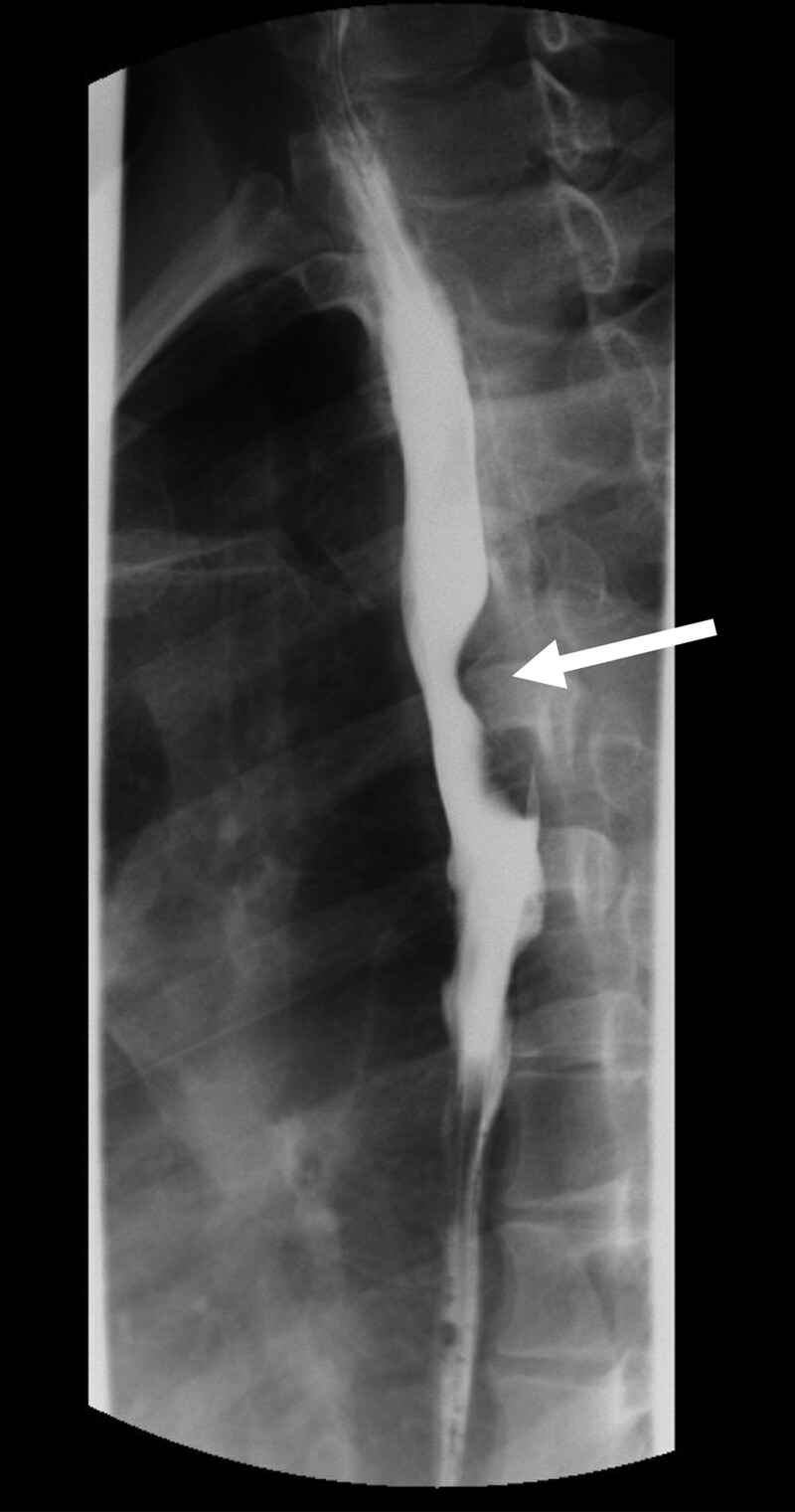
Left anterior oblique esophagogram showing external compression of the posterior wall of the thoracic esophagus at the level of the aberrant right subclavian artery, which caused luminal narrowing (arrow).

A 34-year-old woman who was a nonsmoker and non-alcohol drinker without any significant medical history, including vocal abuse, trauma, intubation, thyroid disease, or surgery, presented to our otolaryngology outpatient department because she had experienced hoarseness and mild dysphagia for 1 month. She also reported discomfort in the throat. No dyspnea, stridor, goiter, or cervical lymphadenopathy was noted upon physical examination. Upon stroboscopic examination, left vocal cord incomplete paralysis was observed. The left vocal fold was only slightly mobile, and some muscular tonus and mucosal waves were noted although reduced (Fig. 1). Chest X-ray revealed no significant abnormalities. The biochemical tests and whole blood cell count were within the normal ranges. Contrast-enhanced computed tomography (CT) of the neck showed an aberrant right subclavian artery arising from the left aortic arch and following a retroesophageal course (Fig. 2), which was causing focal compression of the esophagus (Fig. 3) and, potentially, compression of the left recurrent laryngeal nerve with subsequent left vocal cord paralysis. The CT revealed no aneurysmal dilatation at the orifice of the aberrant right subclavian artery. Features indicating left vocal cord paralysis were present. They included left pyriform sinus dilatation, left aryepiglottic fold medialization, and the sail sign (Fig. 4). An esophagogram showed mild luminal narrowing at the upper third of the esophagus with a smooth contour and an obtuse marginal angle, indicating an external compression lesion (Fig. 5), which was consistent with the location of the aberrant right subclavian artery on CT.

**Figure 6.**
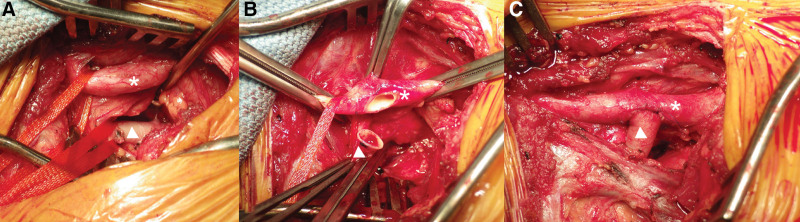
Intraoperative image revealing (A) the right common carotid artery (asterisk) and the aberrant right subclavian artery (triangle) and (B and C) the proximal portion of the aberrant right subclavian artery being oversewn; while the distal portion (triangle) being anastomosed to the right common carotid artery (asterisk).

**Figure 7.**
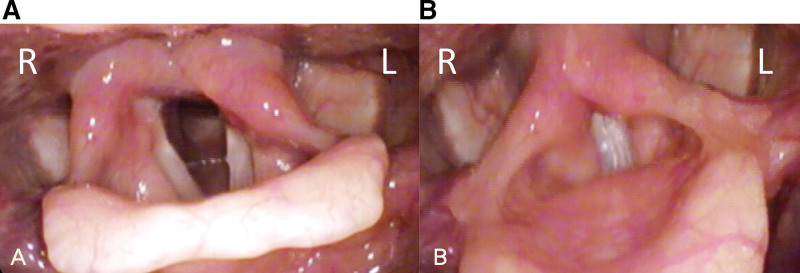
(A) No vocal cord adduction noted. (B) Improvement of left vocal cord incomplete paralysis.

The patient was referred to a cardiovascular surgeon, was admitted, and underwent retroesophageal right subclavian artery resection and right common carotid artery–right subclavian artery bypass (Fig. 6). Steroid treatment was administered after the surgery. After discharge, speech therapy was arranged. The patient underwent regular follow-up at the otolaryngology outpatient department, and significant improvement of the left vocal cord paralysis and no vocal cord adduction were observed upon stroboscopic examination after 3 months (Fig. 7). Moreover, the hoarseness and dysphagia symptoms subsided. Over the 5-year follow-up period, the patient remained well, and no signs of recurrence were noted.

## 3. Discussion

Hoarseness usually results from immobilization of the vocal cords, which can be classified as complete or incomplete vocal paralysis. Common causes of this immobilization include malignant neoplasms, trauma, surgery, inflammation, and idiopathic problems. Complete vocal paralysis occurs when all motor units of the laryngeal nerve are affected, as in the case of surgical resection. By contrast, in incomplete vocal paralysis, only some motor units are affected by compression or straining of the nerve. The recurrent laryngeal nerve on the left side is particularly vulnerable to paralysis due to its longer course than that on the right side. According to Loughran et al, the most common cause of unilateral recurrent laryngeal nerve paralysis is lung malignancy, accounting for 43% of all cases, whereas idiopathic causes, including OS, account for only 11%.^[4]^

**Figure 8.**
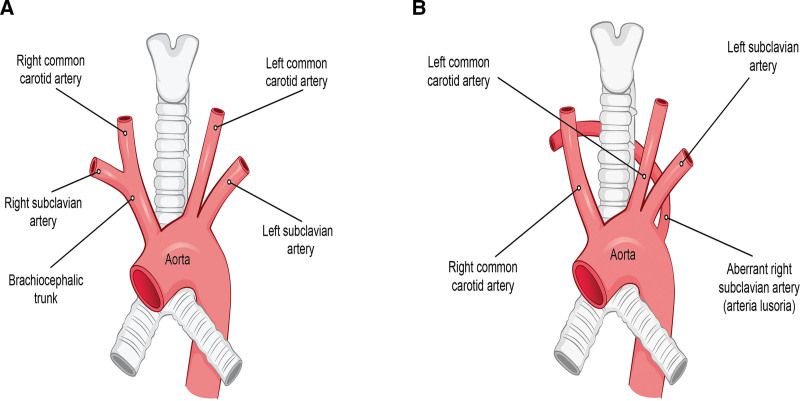
(A) Normal course of the right subclavian artery: originating from the brachiocephalic trunk. (B) Aberrant right subclavian artery: originating from the last branch of the aortic arch.

OS resulting from an aberrant right subclavian artery without aneurysm formation is rare. To our knowledge, only 1 case, by Girardi et al,^[1]^ has been published. The case involved a 70-year-old woman with hoarseness caused by an aberrant right subclavian artery. An aberrant right subclavian artery is the most common embryonic abnormality of the aortic arch, with a prevalence rate of 0.5% to 1.8%.^[5]^ An aberrant right subclavian artery usually originates from the last branch of the aortic arch (Fig. 8) and follows a retroesophageal course to cross the midline, thereby compressing the esophagus and leading to dysphagia. The resulting condition, termed dysphagia lusoria, was first described by David Bayford in 1761.^[6]^

A third of patients with dysphagia lusoria are symptomatic, and dysphagia is observed in 90% of such cases.^[7]^ However, hoarseness, which occurred in the present case, is less common. Only when the aberrant artery compresses the left recurrent laryngeal nerve and causes paralysis are the criteria for OS met. Stroboscopic examination can provide useful information about the vibration ability and abnormalities of the vocal cord. CT is the preferred imaging modality for differential diagnosis. Cross-sectional images can reveal the aberrant branch arising from the distal left aortic arch rather than coursing rightwards.^[7]^ An esophagogram or a barium swallow can reveal indentation on the posterior esophageal wall by the aberrant artery.^[8]^ The aforementioned characteristics are useful for diagnosis. A case of an aberrant right subclavian artery with a retroesophageal course simultaneously compressing the esophagus, which leads to dysphagia lusoria, and the left recurrent laryngeal nerve, which causes hoarseness, has not been published previously. Recognizing this syndrome is crucial for otorhinolaryngologists because it is one of the few syndromes with symptoms of hoarseness and dysphagia and is uncommon. Symptomatic patients should be referred to a cardiovascular surgeon for evaluation and further management, whereas asymptomatic patients should be followed up regularly. Early and accurate diagnosis by an otolaryngologist is essential. This can be aided by the accurate identification of clinical manifestations and imaging features.

## 4. Conclusion

OS caused by an aberrant right subclavian artery following a retroesophageal course without aneurysm formation is rare. This case report may be useful for otolaryngologists, and OS should be considered when patients with both hoarseness and dysphagia are being given a diagnosis.

## Author contributions

**Resources:** Shih-Lun Chang, Nan-Chun Wu.

**Supervision:** Shih-Lun Chang.

**Writing – original draft:** Yen-Wen Chen.

**Writing – review and editing:** Yen-Wen Chen, Shih-Lun Chang, Yun-Ju Shih.
